# Automating insulin delivery through pump and continuous glucose monitoring connectivity: Maximizing opportunities to improve outcomes

**DOI:** 10.1111/dom.15920

**Published:** 2024-09-18

**Authors:** Ananthi Anandhakrishnan, Sufyan Hussain

**Affiliations:** ^1^ Department of Diabetes, School of Cardiovascular, Metabolic Medicine and Sciences King's College London London UK; ^2^ Department of Diabetes and Endocrinology Guy's & St Thomas' NHS Foundation Trust London UK; ^3^ Institute of Diabetes, Endocrinology and Obesity King's Health Partners London UK

**Keywords:** continuous glucose monitoring, CSII, insulin pump therapy, type 1 diabetes, type 2 diabetes

## Abstract

The development of automated insulin delivery (AID) systems, which connect continuous glucose monitoring (CGM) systems with algorithmic insulin delivery from an insulin pump (continuous subcutaneous insulin infusion, [CSII]), has led to improved glycaemia and quality of life benefits in those with insulin‐treated diabetes. This review summarizes the benefits gained by the connectivity between insulin pumps and CGM devices. It details the technical requirements and advances that have enabled this, and highlights the clinical and user benefits of such systems. Clinical trials and real‐world outcomes from the use of AID systems in people with type 1 diabetes (T1D) will be the focus of this article; outcomes in people with type 2 diabetes (T2D) and other diabetes subtypes will also be discussed. We also detail the limitations of current technological approaches for connectivity between insulin pumps and CGM devices. While recognizing the barriers, we discuss opportunities for the future.

## Abstract 2

Answer questions and earn CME credit

## 1 INTRODUCTION

Despite advances in the management of type 1 diabetes (T1D), the daily decision‐making burden in the pursuit of optimizing glycaemia is significant. Therefore, it is not surprising, that for many living with T1D, glycaemia remains above the recommended targets. In the United States, data from the 2018 Diabetes Exchange registry reports that only 17% of children and 21% of adults with T1D achieve their target HbA1c levels^1^ and, in the UK, 2021‐2022 National Diabetes Audit data highlights that almost two‐thirds of adults with T1D do not meet conservative treatment targets for their condition.^2^

Establishing connectivity between continuous glucose monitoring (CGM) devices and insulin pumps (continuous subcutaneous insulin infusion [CSII]), leading to the development of closed‐loop ‘artificial pancreas’ systems, has transformed the management of, and living with, T1D. It is now extending its reach to those with type 2 diabetes (T2D) and other forms of diabetes. Advances in technology will offer further opportunities for connectivity, but they will also present some challenges. There are risks of already evident widening disparities in access.

The aim of the current article is to provide a narrative review that reports on how the connectivity between CGM devices and insulin pumps have benefitted people with diabetes. The historical perspective provided sets the scene for the development of connectivity for automated insulin delivery (AID) systems that may be less known or understood by healthcare professionals (HCPs). It also actively addresses current limitations. Overcoming some of the challenges will maximize opportunities to ensure that these systems offer benefits to the widest number of individuals. We provide an overview of outcomes from T1D trials to highlight how outcomes have been improved, with a further focus on diabetes subtypes other than T1D.

## 2 BACKGROUND

### 2.1 Initial insulin pumps and closed‐loop insulin delivery

In 1964, Arnold Kadish used an ‘on–off’ control system to connect continuous real‐time intravenous glucose measurements to two intravenous syringe pumps containing insulin and either glucose or glucagon to maintain glycaemia within a set target range.^3^ Although Kadish's system did not utilize algorithmic titration of insulin or glucagon delivery, it is widely credited as the first automated, or ‘closed‐loop’, insulin delivery system. Ten years later, two groups in parallel established communications between intravenous glucose measurements and algorithmic insulin delivery: Albisser's group in Canada^4^, ^5^ and Pfeffier's group^6^ in Germany developed systems where an algorithm hosted on a microcomputer titrated intravenous insulin and/or glucose delivery depending on intravenous blood glucose levels. Pfeffier's group's system was commercialized as the Biostator^7^ in 1977, although its size and complexity meant that its use was largely confined to research.

Work in Paris in 1974 by Slama et al.^8^ explored wearable continuous insulin delivery. Insulin was delivered ‘open‐loop’ at predetermined basal rates and 15‐fold higher prandial rates and infused via an intravenous insulin pump worn in a shoulder bag by seven participants with T1D over a trial period of 1‐5 days. Compared with three times per day subcutaneous insulin, the results reported positive glycaemic outcomes. The first portable AID or ‘closed‐loop’ system was developed by Shichiri and colleagues in Japan in 1984 using two intravenous pumps for insulin and glucagon, and a subcutaneous glucose sensor connected by a microcomputer‐hosted algorithm.^9^ Trialled in three people with T1D (pwT1D) and seven without diabetes, the system showed superiority compared with multiple daily injections (MDI) or ‘open‐loop’ insulin delivery. The practicalities and safety of intravenous insulin delivery limited the wider utilization of such technologies. To address such limitations, Keen, Pickup and colleagues from Guy's Hospital London began work on continuous subcutaneous insulin (insulin pump/CSII) delivery. The outcome of their research showing the feasibility of CSII in 12 participants with insulin‐dependent diabetes was published in 1978.^10^ Following publication, during the next 4 years, several groups reported the safety of CSII use in pwT1D in inpatient^11^, ^12^ and short‐^13^, ^14^ and long‐term^12^, ^15^, ^16^ outpatient settings. This led to the development and commercialization of the first body‐worn insulin pump in 1983.^17^, ^18^

The earliest commercial insulin pumps were large devices infusing insulin via metal needles with a comparatively short battery life, little flexibility in the rate of insulin delivery^19^ and limited safety alarms. Hence, the complications of hyperglycaemia and diabetic ketoacidosis were not uncommon.^20^, ^21^, ^22^, ^23^ The 1990s and 2000s saw a revolution in the development of insulin pumps. Safety features such as alerts for infusion set occlusion and low battery or low insulin reservoir were integrated alongside the miniaturization of pump devices, longer‐life batteries and plastic catheter infusion sets.^19^

### 2.2 CGM development

With advances in pump device technology, the next limiting step for wider practical development and adoption of closed‐loop systems became refinements in real‐time interstitial fluid glucose sensing via CGM devices. The first commercially available CGM device was developed by Minimed (now Medtronic) in 1999 and consisted of a cable physically connecting the sensor and its receiver. While it enabled the recording of glucose values over 3 days, data were not available in real time for the user. Manual download onto a computer via a ‘communications cradle’ was performed by a healthcare provider for retrospective analysis.^24^ This lack of real‐time connectivity presented another obstacle to the consideration of a practical AID device.

In 2004, Medtronic's Guardian REAL‐Time CGM device allowed real‐time programmable high‐ and low‐glucose alerts, features that have subsequently become industry standard. In the same year, Dexcom launched the short‐term sensor, its first CGM device allowing real‐time glucose review; this was followed, in 2008, by Abbott's real‐time CGM device, the freestyle navigator.^25^ These first‐generation CGM devices all necessitated a battery‐operated secondary receiver for data review.^26^, ^27^, ^28^ Medtronic's Guardian REAL‐Time also introduced wireless radiofrequency (RF) connectivity between sensor and receiver, and the subsequent development of data transmitter accessories such as the Dexcom SHARE enabled viewing CGM data on handheld devices via wireless upload of receiver data onto the cloud.^25^, ^26^ The need for a distinct CGM receiver was removed after Dexcom released the G5, which enabled direct connectivity between the CGM device and smartphone application.^29^ Smartphone connectivity, with options for handheld readers, is now becoming the norm for currently available CGM devices. The majority of CGM options are real time, where interstitial glucose levels are continuously transmitted to a receiver via RF or Bluetooth. The alternative option is intermittently scanned CGM (isCGM). With isCGM, users scan the sensor to obtain readings via near‐field communication (NFC). The continuous wireless data transfer of realtime CGM (rtCGM) devices facilitates their use in AID systems whilst the requirement for manual scanning in the commercial use of isCGM devices means their adoption within AID systems is impractical. However, the potential to co‐opt wireless NFC to enable automated intermittent glucose measurements from isCGM devices may allow the integration of these devices within AID systems.

### 2.3 The call for connectivity

The development of smaller, smarter CGM devices and insulin pumps in the 2000s^30^ saw the focus shift towards establishing connectivity between systems. In 2006, the Juvenile Diabetes Research Foundation (JDRF) established the Artificial Pancreas Project targeted towards the development of AID systems.^31^ In 2009, a roadmap to achieving the artificial pancreas comprising six stages of automation was proposed^32^: stages one to three described systems targeting hypoglycaemia; stage four consisted of hybrid closed‐loop (HCL) and stage five fully closed‐loop (FCL) systems; with stage six being a fully automated multihormonal closed‐loop system.

The earliest phase of modern connectivity saw the development of low‐glucose suspend (LGS) devices that suspend insulin delivery when glucose levels fall under a set threshold,^33^ commercialized in 2009 (CE Mark approval of the MiniMed Veo System^34^). The evolution of LGS systems saw the development of predictive LGS (PLGS) systems that halt insulin delivery in preparedness for hypoglycaemia, and have been in use since early 2015 (MiniMed 640G CE mark January 2015).^34^, ^35^, ^36^ This was followed by the development of HCL systems. HCL systems connect rt‐CGM devices with algorithmic insulin delivery from an insulin pump. The algorithm constantly alters insulin delivery via the pump based on regular CGM‐derived glucose readings taking into consideration other factors such as current glycaemia, predicted glycaemia and insulin on‐board. The first HCL systems required manual connectivity between the sensor, algorithm and pump; manual input of sensor glucose values into a computer‐based algorithm with subsequent manual adjustment of basal insulin infusion rate according to algorithmic calculation necessitated.^37^ The adoption of wireless communication between sensor, algorithm and pump has facilitated wider use of HCL systems, with glycaemic and quality of life (QoL) benefits reported both from clinical trials and real‐world evidence (RWE). Nevertheless, there are still challenges concerning connectivity that need to be overcome.

## 3 TECHNICAL ADVANCES ENABLING CONNECTIVITY BETWEEN DIABETES DEVICES

Connectivity between the three limbs of an AID system is key in enabling optimal glycaemic control. The development of the first HCL systems began in tandem with LGS technology.^38^ However, delays to commercialization saw the birth of the open‐source (OS) closed‐loop movement.^39^ Alongside the #WeAreNotWaiting, in 2013, a group of pwT1D and their families collaborated to develop OS closed‐loop algorithms to respond to the unmet need for commercial HCL systems. The first OS HCL systems used a hardware radio ‘bridge’ to communicate between the pump and algorithm because of the built‐in RF communication of older insulin pumps.^39^, ^40^ The first OS AID systems implemented LGS technology and subsequent versions were further developed into HCL systems. In 2016, Medtronic's MiniMed 670G became the first commercially available HCL system^41^ utilizing RF to communicate between a rt‐CGM sensor and closed‐loop algorithm housed within the insulin pump. The later adoption of Bluetooth technology within commercial insulin pumps facilitated the housing of commercial and OS closed‐loop algorithms within secondary handheld mobile devices. Table 1 provides details of the currently available commercial and OS HCL systems with their connectivity considerations.^40^, ^42^

**TABLE 1:** Commercial and open‐source AID systems.

System	Algorithm name	Algorithm type	User interface	Compatible CGM device	Compatible insulin pump	Connectivity
Open source						
Open APS	Oref0^43^	Insulin dosing based on a number of scenarios that it forecasts with different types of predictions^48^	Android and Apple smartphones Any smartwatch	Dexcom G4, G5, G6; Medtronic Real‐Time Revel and Enlite; other systems via Nightscout^a^	Medtronic 512/712, 515/715, 522/722, 523/723, 554/754	Radiofrequency 900 mHZ
AndroidAPS	Uses Open APS algorithm with modifications		Android smartphone ‘Wear OS’ Google smartwatch	Dexcom G4, G5, G6; FreeStyle Libre, Libre 2; Eversense; Medtronic Guardian 2; Medtrum A6; PocTech; Gluco24	AccuChek Spirit Combo; AccuChek Insight; Dana R or RS; Medtronic 512/712, 515/715, 522/722, 523/723554/754 OmniPod Eros	Radiobridge; Classic Bluetooth; BLE
Loop	Loop	MPC	Apple smartphone Apple smartwatch	Dexcom G4, (with Dexcom SHARE) G5, G6, G7; Medtronic Enlite 2; FreeStyle Libre with third party software, Freestyle Libre 2	OmniPod Eros; Medtronic 515/715, 522/722, 523/723, 554/754	Radiofrequency 916 MHz; BLE
iAPS^44^	Uses Open APS algorithm with modifications		Apple smartphone Apple smartwatch	Dexcom G5/G6/ONE/G7 Glucose Simulator Libre Transmitter Glucose Direct Medtronic Enlite Other systems via x‐drip^b^ and Nightscout^a^	Omnipod DASH pumps Omnipod Eros pumps Medtronic 515/715, 522/722, 523/723, Medtronic Worldwide Veo 554/754, Medtronic Canada/Austraian Veo 554/754	RileyLink or similar needed for Omnipod Eros Bluetooth for Omnipod DASH
Trio^45^	Uses Open APS algorithm with modifications		Apple Smartphones Apple Smartwarch	Dexcom G5/G6/ONE/G7 Glucose Simulator Libre Transmitter Glucose Direct Medtronic Enlite Other systems via x‐drip^b^ and Nightscout^a^	Omnipod DASH pumps Omnipod Eros pumps Medtronic 515/715, 522/722, 523/723, Medtronic Worldwide Veo 554/754, Medtronic Canada/Austraian Veo 554/754 DanaRS (in the future)	RileyLink or similar for Omnipod Eros Bluetooth for Omnipod DASH
Commercial						
Medtronic 670G/770G	SmartGuard	PID	Insulin pump 770G: Android or Apple phone (view only)	Guardian Link 3	Medtronic 670G/770G	Radiofrequency 2.4 GHz
Medtronic 780G	SmartGuard	PID and FL	Insulin pump Android or Apple phone (view only)	Guardian Link 4	Medtronic 780G	BLE
Tandem Control‐IQ	Control IQ 1.0	PID	Insulin pump Android or Apple phone app (t:connect mobile in the United States for mobile view and bolus)	Dexcom G6	Tandem T:Slim x2	BLE
CamAPS Fx	CamAPS FX	MPC	Android phone	Dexcom G6 Freestyle Libre 3	DANA RS; Dana‐I; Ypsopump	BLE
Diabeloop DBLG1	DBLG1	MPC	Separate portable device or Android or Apple phone app	Dexcom G6	Kaleido patch AccuCheck Insight; Dana‐I; EOFlow	BLE
Omnipod 5	SmartAdjust	MPC	Controller or app available for certain android smartphones	Dexcom G6 Freestyle Libre 3	Omnipod 5 patch pump	BLE
Medtrum touchcare Nano^40^, ^42^	Artifical Pancreatic Algorithm		Separate portable device or Android or Apple phone app	Touchcare Nano CGM	Medtrum Easy Patch	BLE
I‐let bionic pancreas system^46^, ^47^		MPC	Android or Apple phone app	Dexcom G6 or DexcomG7	iLet insulin pump	Bluetooth

*Note*: Information sourced from Braune et al.^40^ unless otherwise detailed.

Abbreviations: AID, automated insulin delivery; BLE, bluetooth low energy; CGM, continuous glucose monitoring; FL, fuzzy logic; MPC, model predictive control; PID, proportional integrative derivative.

^**a**^ Nightscout is an open‐source project that allows real time access to a CGM and insulin pump data via cloud upload.

^b^ x‐drip is an Android app that collates data from CGM devices and insulin pumps to be uploaded to Nightscout.

Communication dropouts between components of HCL systems may result from connectivity being interrupted by physical barriers, interference from another wireless device, or inadvertent disabling of wireless connectivity on the algorithm‐hosting device. Communication errors may also occur between either the insulin pump or rtCGM device and platforms designed to receive the data upload; these are secondary to issues related to data (internet or cellular), Bluetooth connectivity or incorrect login details on mobile‐based apps. Battery life, whether in the CGM transmitter, insulin pump or mobile device, could also pose a barrier to connectivity.^43^

### 3.1 Radiofrequency communication

RF waves are a subgroup of electromagnetic waves that transmit data wirelessly within a broad frequency (typically 3 kHz‐300 GHz) and distance range. The lower the frequency, the wider the transmission range, but the lower the rate of data transfer.^49^ RF waves are utilized by electrical devices for communication (between one or more antennae and receivers located on separate devices) and for non‐communicative purposes. Because of their large communication range, devices operating via RF can consume significant energy, affecting battery life. This substantially impacts battery requirements, as well as the weight and size of devices.

The industrial, scientific and medical (ISM) radio bands are internationally designated parts of the RF spectrum reserved for use by ISM fields, with the exclusion of telecommunication devices. Although ISM bands vary between countries, certain bands are shared, one of which is 2.4 GHz.^50^, ^51^ The 2.4‐GHz ISM radio band was utilized to enable older generation insulin pumps to deliver remote boluses through a separate handheld device.^52^ This device also functioned as a CGM receiver for the MiniMed 630G with a Contour Next Link 2.4 meter,^52^ or as a standalone remote controller for the MiniMed MMT‐500 or MMT‐503 controllers used with Medtronic's MiniMed 508 or MiniMed Paradigm families of insulin pumps.^52^, ^53^ However, the RF communications between the MiniMed remote controller systems were found to pose cybersecurity risks with the potential for unauthorized access to, and control of, insulin delivery. This led to an urgent discontinuation and device recall of these systems by Medtronic in 2018.^53^, ^54^ It was the utilization of such loopholes to secure wireless RF connectivity that facilitated the development of early versions of OS closed‐loop systems. In the commercial world, Medtronic initially used the 2.4‐GHz frequency ISM band for wireless communication in its 640G^55^ and 670G^56^ AID systems, later transitioning to the use of Bluetooth low energy (BLE) in its most advanced HCL system, the MiniMed 780G.^57^

### 3.2 Bluetooth low energy

The designation of ISM bands aimed to mitigate interference between wireless communications from ISM and telecommunication devices. However, increasing congestion of the RF spectrum has seen wireless communication systems, including Wi‐Fi and Bluetooth, making use of the 2.4‐GHz ISM RF band.

Bluetooth is a form of wireless technology that operates via 2.4‐GHz RF waves and connects digital devices over short distances. Today's Bluetooth technology includes classic Bluetooth and BLE.^58^, ^59^ BLE is increasingly used in healthcare technology because of its reduced power consumption compared with classic Bluetooth and ISM RF bands. BLE operates via periodic data streaming between communicating devices that become simultaneously active from dormant states.^59^ Although this intermittent transfer is not compatible with phone conversations or streaming videos, it provides a low‐power option for systems that exchange intermittent data such as HCL systems, which cycle their closed‐loop communication between the rtCGM device, algorithm and insulin pump every 5‐12 minutes, depending on the system.^60^ Significant changes in glucose levels are unlikely to occur during the short intervals between periodic data transfers via BLE‐utilizing HCL systems. This feature, however, could serve as a contingency measure to accommodate for communications not received because of interruptions in connectivity.

HCL systems communicating via Bluetooth can host algorithms in a device distinct from the insulin pump, although proximity to this secondary device is needed. In those using patch pump systems, this secondary device provides both glycaemic and connectivity alerts (provided by either an insulin pump or a secondary handheld device in tubed systems).

### 3.3 Interference

**FIGURE 1:**
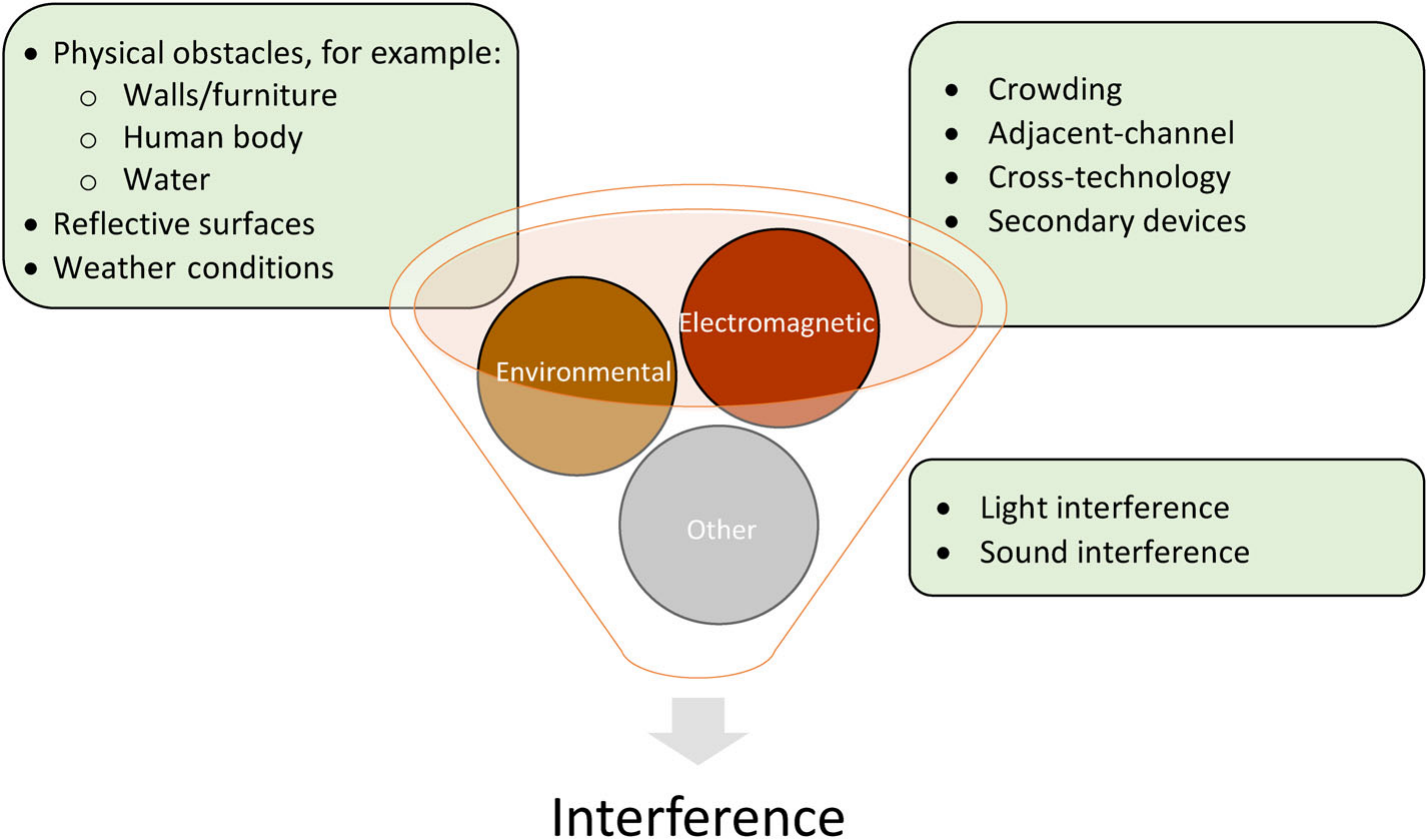
Interference to wireless connectivity; attenuation of Bluetooth signal strength occurs naturally with distance and is also influenced by interference that can be broadly classed as environmental or electromagnetic. Environmental obstacles between the transmitter and receiver can degrade connectivity and include physical barriers like walls, objects, the human body and changes in transmission media from water to air. Reflective surfaces that scatter radio waves and humidity also cause interference.^49^ Electromagnetic interference can be caused by communicative or non‐communicative devices operating via radiofrequency. Sound waves can cause interference to other sound‐producing devices and light transmission can interfere with communication systems transmitting signals through other frequencies.^62^

Interference in wireless communication is the process by which communication between a transmitter and its receiver is interrupted or weakened. Interference can be electromagnetic, environmental or can come from other sources (Figure 1).^61^, ^62^ Real‐world outcomes with the first commercial closed‐loop system, the Medtronic 670G,^63^ reported that 62% of those discontinuing after 1 year cited sensor issues, including unplanned system exits from auto mode, as reasons for closed‐loop discontinuation. Findings suggest the quality of wireless connectivity between limbs of a closed‐loop system to be a key factor in determining device usability. Because interference affects the quality of wireless transmission, reducing interference is probable to increase the reliability, thus the usability, of closed‐loop systems.

Wireless connectivity requires a clear line of sight between transmitter and receiver. For HCL systems housing interface and algorithms within an insulin pump and receiving communications from a CGM device, a clear line of sight may be maintained, but electromagnetic interference (EMI) can still cause connectivity issues leading to additional frustration and device burden. EMI can occur because of crowding, where the number of wireless signals that share the same frequency cause interference to communications within one or more systems.^64^ Adjacent‐channel interference is another form of EMI that occurs when devices operating at overlapping frequencies cause interference to communications within one or more systems.^62^ Cross‐technology interference arises when different wireless technologies using the same frequency waves (such as ISM bands, Wi‐Fi and Bluetooth operating in the 2.4‐GHz frequency) cause interference to communications within one or more systems.^65^ EMI from devices like microwave ovens (that operate at 2.4‐GHz frequency), mobile phones and airport security systems^66^ may also occur, the latter being especially relevant to HCL systems, with the extent of interference varying between manufacturers.

Environmental interference includes weather conditions like rain and humidity that can affect wireless transmission and physical barriers that can weaken signals or distort the line of sight between transmitter and receiver. Such barriers include water, which is known to attenuate wireless signalling, although recent data have shown that the Omnipod 5 (OP5) patch pump, which utilizes BLE, maintains connectivity during submersion in water for interdevice distances of up to 13 cm.^67^ The human body can also act as a source of environmental interference, with human tissue able to absorb radiation at the 2.4GHz frequency.^68^ A recent experiment exploring Bluetooth signal attenuation through human body tissue analogues showed attenuation from muscle tissue to be greater than analogues of water or fat tissue^68^; with regards to clinical practice, alterations in HCL connectivity may be impacted by user body composition. Absorbability by human tissues may also raise safety concerns regarding the use of Bluetooth on wearable medical devices. However, Bluetooth technology has a power output well within the limits specified by the International Commission on Non‐Ionizing Radiation Protection, and thus is generally considered to be safe.^68^

**FIGURE 2:**
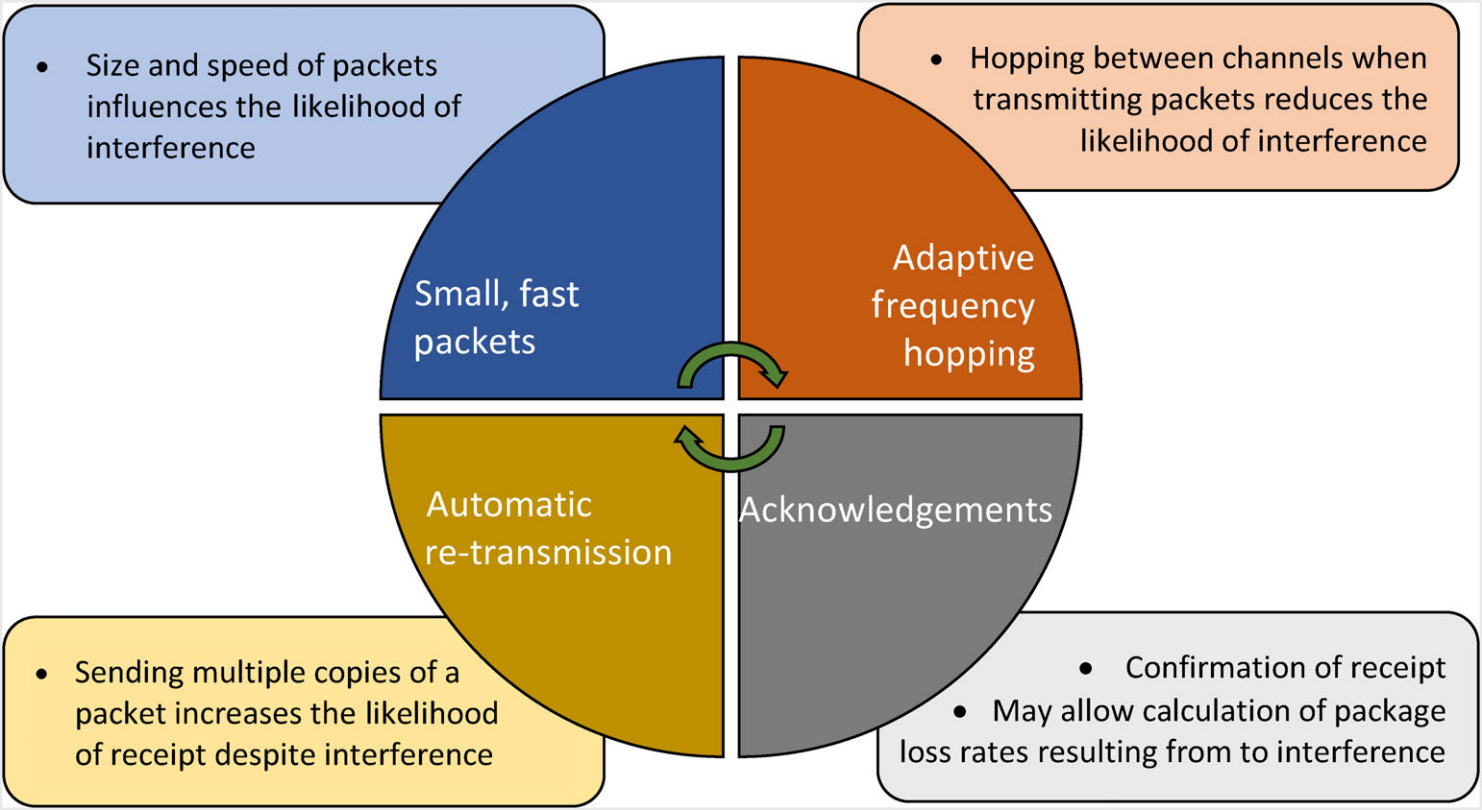
Methods used by Bluetooth technology to maintain connectivity; packets refer to small portions of larger data. Clockwise: adaptive frequency hopping is the process of dynamically switching between communication channels to reduce the likelihood of interference; despite interventions to prevent interference, data loss can still occur; acknowledgements confirm receipt of packages. Automatic re‐transmission is the process of sending multiple copies of data packets to reduce the likelihood of signal contamination by interference. Smaller, faster packets reduce the likelihood of data being intercepted by interfering signals.^69^

Interference to connectivity within HCL systems may have safety implications, and although interference from HCL systems to secondary devices is less of a safety concern, it can cause day‐to‐day frustrations, and interference between HCL and other medical electrical equipment is also possible. Strategies for mitigating interference include increasing the distance between, or turning off interfering external devices,^56^, ^57^ although the practicality of achieving this may prove challenging at times. Bluetooth technology employs a number of techniques to mitigate EMI (Figure 2).^69^ One such technique is known as adaptive frequency hopping (AFH). Bluetooth enables AFH by dividing its frequency band into smaller channels (40 for BLE and 79 for classic Bluetooth), and rapidly hops between these channels during signal transmission. This reduces the likelihood of occupying a given frequency at a given time. To further minimize interference, Bluetooth adjusts its hopping sequence dynamically and monitors and avoids channels that are noisy or busy.^69^, ^70^

### 3.4 Cybersecurity

Despite the benefits of wireless connectivity between CGM devices and insulin pumps, the increased utilization of wireless connectivity brings with it added cybersecurity concerns. Devices operating via RF and Bluetooth offer authentication and encryption that act together to create secure connectivity between communicating devices.^71^ However, both are optional features, so mandating their use may assist in preventing unauthorized data access. Devices operating via Bluetooth announce when they are available for pairing. This can be detected by other devices within the proximity. Although device authentication can prevent an individual's personal device pairing with unknown devices, the user could be identified during announcement, and unauthorized access to data whilst authenticated devices are announcing themselves available for pairing is not impossible. Switching devices to ‘not discoverable’ modes may protect against such risks without affecting connectivity between authenticated paired devices. In those HCL systems housing algorithms within smartphone applications (apps), the possibility of security breaches from corrupt mobile downloads or installed apps is also possible.^71^ The use of multifactor authentication to access app data may mitigate this risk.

The ability of OS systems to incorporation newer insulin pumps utilizing Bluetooth connectivity (Table 1) proves that even advancing methods of wireless connectivity are not infallible. Identifying means to mitigate cybersecurity breaches may facilitate user safety and trust within commercial systems.

### 3.5 Cloud upload

**FIGURE 3:**
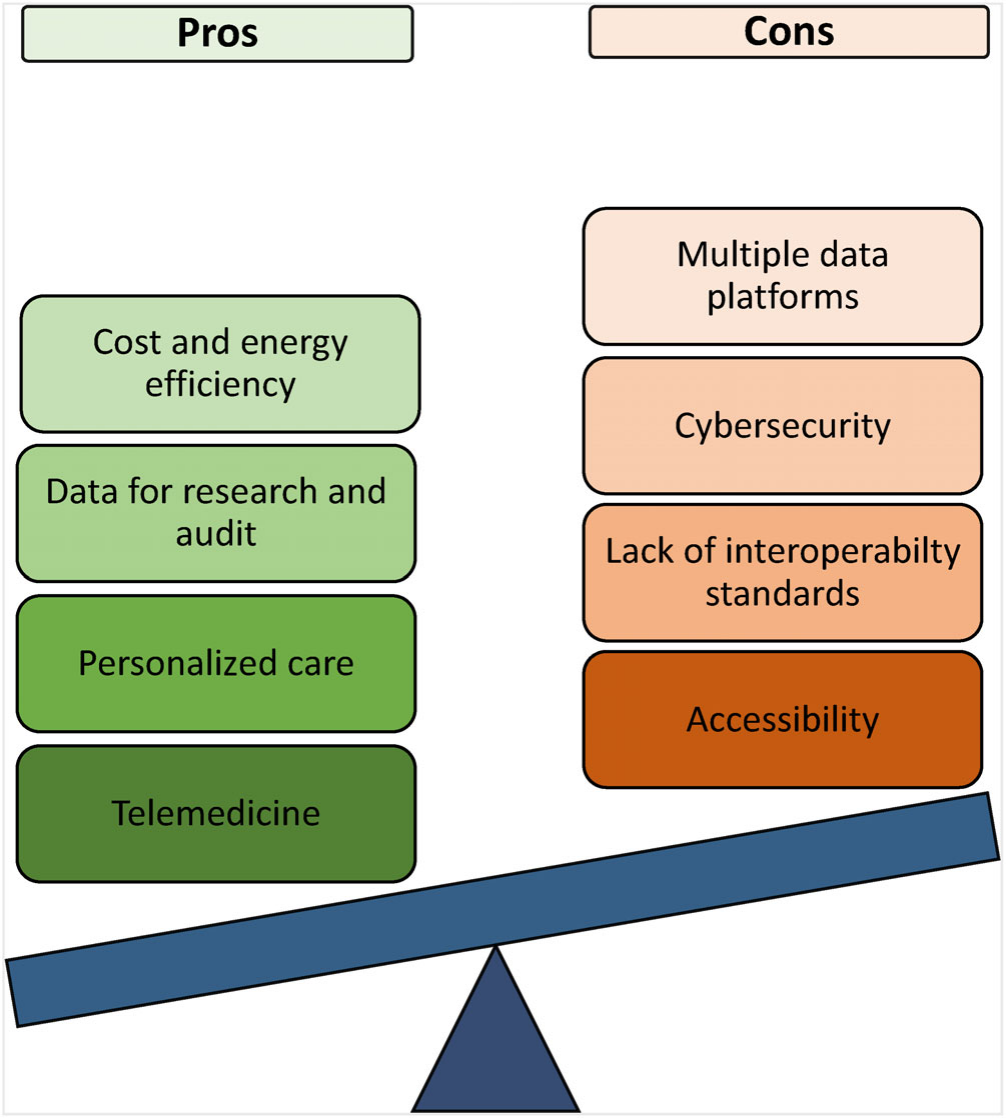
The pros and cons of automated wireless upload to the ‘cloud’; the advantages of cloud upload include automated access to glucose data, removing the need for manual glucose diaries, benefitting those with diabetes by reducing the burden of management and HCPs by allowing access to large volumes of datasets. The cost and energy benefits, ability to facilitate personalized care and telemedicine, and being a broad data source for research and audit, are other advantages of using automated wireless upload to the cloud. Limitations come from the necessity for HCPs to access multiple data platforms to access data from patients using different technologies. Similarly, for people with diabetes when transitioning from continuous glucose monitoring alone, a sensor augmented pump or open‐loop to hybrid closed‐loop disconnection from previous data platforms with download of new data platforms and/or smartphone software is needed. The pre‐existing platform remains active, however, and although data‐protection processes are in place, these are not immune to security breaches. A lack of interoperability standards and the need for internet connectivity limiting accessibility are other limitations of automated wireless upload of glucose data to the cloud. HCP, healthcare professional.

Many HCL systems now automatically upload data to the cloud. Remote access to data has been shown to be important to pwT1D^72^ and their carers,^73^ with the potential to facilitate personalized clinical consultations^74^ and telemedicine.^75^ In addition, the obviated need for software and hardware for data upload and storage could also provide cost‐ and energy‐saving benefits. The available data stored on the cloud are also accessible to HCPs and relevant stakeholders, forming a useful resource for audit and research, with the potential to impact outcomes for broad populations. Access to broad datasets may also facilitate the development of artificial intelligence (AI)‐assisted algorithmic insulin delivery, but careful legislation will be required to ensure consensual responsible use of data. To this end, ensuring the confidentiality of the user, the integrity of uploaded data, and clear establishment of the ownership and privacy of healthcare information are key security considerations when it comes to the storage of cloud‐based data;^76^ the latter preventing unauthorized access without approval from all relevant stakeholders. It must also be remembered that the ease and efficacy of access to systems operating via cloud‐based systems depend on the strength and availability of internet connection,^76^ and not all people with diabetes and/or their carers may have access to the technology required. Figure 3 illustrates the advantages and disadvantages of using the cloud.

## 4 CURRENTLY AVAILABLE SYSTEMS

Currently available HCL systems are grouped as either commercial or OS systems; both comprise a CGM device, insulin pump and algorithm. While currently available commercial HCL systems are limited to specified pump and sensor combinations, OS systems provide interoperability between a number of commercial insulin pumps and CGM devices and also offer smartwatches as an added user interface^40^ (Table 1).

Five OS AID systems are most commonly in use in the OS closed‐loop community: (i) OpenAPS communicates via RF^48^; (ii) AndroidAPS uses the OpenAPS algorithm, on an Android smartphone, communicating via radio‐bridge, RF, Classic Bluetooth or BLE; (iii) Loop, on an Apple iPhone smartphone or smartwatch, communicating via RF or BLE^40^; (iv) iAPS^44^; and (v) Trio,^45^ both on an Apple iPhone smartphone or smartwatch using Bluetooth connectivity, or via a separate device that converts the RF communications of older insulin pumps to Bluetooth waves (such as RileyLink).^44^, ^45^ Eight commercially available HCL systems are now approved for use in pwT1D across Europe and North America. Commercial systems host their algorithm within the insulin pump (670G, 780G, Control‐IQ, i‐Let^46^, ^47^), on a separate handheld (OP5) or smartphone device (CamAPS‐FX and Diabeloop, Medtrum Touchcare Nano), and utilize BLE or RF communications.^40^, ^42^ For those systems compatible with specific smartphone devices, the need to purchase a compatible device may limit accessibility, and operating system software updates also have the potential to cause connectivity issues.

### 4.1 Algorithm development

Mathematical models of glucose–insulin interactions have been studied during the last 50 years^77^ with several trialled in silico and in clinical studies.^78^ Three classes of control algorithm are in use by currently available commercial HCL systems. The studies of Hovorka et al.^38^, ^79^ first outlined the model predictive control (MPC) algorithm that titrates insulin delivery based on predicted changes to glycaemia factoring in rtCGM data, insulin administered to date and expected fluctuations in glucose levels, among other variables.^80^, ^81^ The proportional integral derivative (PID) algorithm was first described by Steil et al.,^82^ and it alters insulin delivery by analysing rtCGM glucose measurements against the extent of deviation from target glucose (proportional component), the absolute difference between rtCGM–derived glucose and target glucose (integral component), as well as the rate of change of measured glucose (derivative component).^80^, ^81^ In 2010, the MD‐Logic algorithm using fuzzy logic models to drive insulin delivery was piloted; instead of using mathematical models, insulin delivery is altered based on predicted decisions that would be made by diabetes clinicians in response to detected rtCGM metrics.^83^ The development of further models, including adaptations and future potential to incorporate AI and machine learning (ML), is ongoing. Algorithm details and development from industry and commercial systems remain proprietary, whereas those of OS systems can be accessed publicly.

### 4.2 Processor requirements

Current control algorithms not only receive information from CGM devices and insulin pumps every few minutes, but are adaptive, altering insulin delivery from the experience of their users^84^, ^85^; however, such complex calculations can pose challenges if the algorithms are hosted on devices with low processing power (e.g. insulin pumps or mobile phones).^86^

## 5 CLINICAL BENEFITS IN T1D

CGM and insulin pump devices may be used independently or together as sensor‐augmented insulin pump therapy (SAP). Although CGM and insulin pump devices, improved glycaemic control when compared with MDI^87^, ^88^, ^89^ and insulin pump^90^ alone, their use independently, or in tandem as SAP, does not reduce the management burden of T1D.^91^ When AID systems targeting hypoglycaemia were compared with SAP, LGS devices were shown to improve hypoglycaemia without increases in HbA1c,^33^, ^92^ and PLGS were shown to reduce the severity and duration of hypoglycaemia,^35^, ^93^ with resultant QoL benefits in pwT1D.^94^ However, increased incidences of hyperglycaemia were reported with PLGS systems in a paediatric population.^95^

Glycaemic benefits from trials and RWE in pwT1D have been detailed in recent systematic reviews on commercial HCL systems.^42^ Several meta‐analyses of randomized controlled trials (RCTs) report on the comparative efficacy and safety of HCL systems in pwT1D compared with MDI or SAP.^96^, ^97^, ^98^, ^99^, ^100^, ^101^, ^102^, ^103^ Baseline clinical characteristics were seen to influence outcomes from HCL, where those with the highest baseline HbA1c and lowest time in range (3.9‐10 mmol/L) were seen to have the greatest glycaemic benefits.^103^, ^104^ Further RWE from the UK also highlights benefits in people with higher baseline HbA1c.^105^ Recent studies have also highlighted benefits during pregnancy.^106^, ^107^ Variations in the design of clinical trials impair the ability to perform between‐study comparisons, and the absence of a comparator in single‐arm trials limits interpretation of the relative contributions of HCL use and study effect on observed outcomes.

Outcomes from OS systems have also been extensively collected in real‐world settings.^108^, ^109^ RCTs for OS systems have been more difficult because of regulatory and funding constraints. Nevertheless, the CREATE trial provided data for a multicentre, open‐label RCT for a modified version of AndroidAPS.^110^

### 5.1 Psychological and quality of life benefits in T1D

Real‐world studies and clinical trials of HCL systems in T1D are increasingly including person‐reported outcome measures (PROMs) evaluating QoL changes as outcome measures. A meta‐analysis of reported PROMs reported a trend towards improvements in diabetes‐specific distress and a reduction in hypoglycaemia fear,^111^ with no change in generic QoL outcomes. While not reported consistently, HCL systems may also have positive effects on subjective and objective sleep in pwT1D,^112^ parents of children with T1D^113^ and women with T1D using HCL systems in pregnancy. Reductions in the physical, mental and emotional demands of diabetes in pregnancy were also reported in the latter group.^114^

Qualitative analyses of semistructured interviews further detail the QoL and psychosocial impacts of HCL systems; reductions in the decision‐making burden, as well as feelings of stigmatization and worry were reported in children, young people and adults with T1D, alongside increased flexibility, spontaneity and confidence.^115^, ^116^ Interestingly, qualitative work has shown that, as with glycaemic outcomes with HCL systems, baseline characteristics influence outcomes; those with higher baseline HbA1c tend to report greater satisfaction and trust in HCL systems, while those with better baseline control are more probable to engage in compensatory behaviour and experience greater cognitive and emotional stress in the event of suboptimal auto‐mode performance.^117^ As with glycaemic outcomes, skewed ethnic and socioeconomic representation limits interpretation. Furthermore, lack of a consensus in measuring and reporting PROMs across studies makes it difficult to draw effective comparisons and conclusions.

### 5.2 HCL systems in other diabetes subtypes

The evidence for HCL use in people with type 2 diabetes (pwT2D) is small but expanding (Table 2). One small multicentre outpatient study^118^ and a small single‐arm study^119^ in pwT2D using insulin therapy show the feasibility of HCL systems, but their cohort sizes and study periods limit interpretation. RWE and randomized studies with comparator groups are needed to determine the practicality and efficacy of different HCL systems in pwT2D. Limited small, short‐term studies assessing outcomes of closed‐loop therapies in those with cystic fibrosis‐related diabetes have also been undertaken^120^, ^121^ (Table 2). A larger study is currently being undertaken in the UK.^122^

**TABLE 2:** Clinical trials of closed‐loop systems in T2D and CFRD.

	i. Duration	Closed‐loop intervention	Methods	Primary outcomse	Outcomes measures					Study‐related adverse events	Limitations
	ii. Participants										
	iii. Country				% TIR	% Time below range	% Time above range	Other outcomes	Patient‐related outcome measures		
	iv. Study type										
	v. Key inclusion										
T2D – HCL											
Davis et al. (2023)^1^, ^118^	2 mo 24 adults with T2D United States Retrospective analysis On insulin, no pump use within 3 mo of screening	HCL OP5 with SmartAdjust	Single‐arm multicentre outpatient study comparing AID to insulin pen	△ % Time with sensor glucose ≥ 250 mg/dL and < 54 mg/dL	△70‐180 mg/dL	△ % time < 54 mg/dL	△ % time ≥ 250 mg/dL	△HbA1c −1.3% ± 0.7% *P* < .0001	Increased insulin delivery satisfaction	Nil	Single‐arm study Sample size Study duration
					+21.9 ± 15.2 *P* < .0001	No significant change Pen 0 (IQR 0, 0.06) AID 0 [IQR 0, 0.03] *P* = .4543	−16.9 ± 16.2 *P* < .0001				
Levy et al. (2024)^119^	6 wk 30 adults with T2D United States Prospective single‐arm trial On insulin, no previous Tandem T‐slim use	HCL Tandem T:Slim with Control IQ	Prospective single‐arm study	Not specified	△ 70‐180 mg/dL	△ % time < 54 mg/dL	△ % time > 250 mg/dL	Baseline HbA1c influences TIR changes; greater change in ≥ 8% baseline HbA1c	PROMIS Sleep and DIDS Diabetes Impact score no change Reduced DIDS Device satisfaction score 6 wk from screening *P* < .006	Nil	Single‐arm study Sample size Study duration
					+15% (95% CI 6%, 24%) *P* = .004	No significant change −0.06 (95% CI −0.19, −0.07) *P* = .32	−4.1 (95% CI −7.5%, −0.6%) *P* = –.02				
T2D – FCL											
Thabit et al. (2016)^123^	72 h 40 adults with T2D UK Randomized control trial On insulin +/− other glucose‐lowering agents	FCL Dana‐R with MPC algorithm	Single‐centre, open‐label, parallel‐group inpatient study comparing FCL to standard insulin therapy	△ % Time in target range (5.6‐10 mmol/L [100‐180 mg/dL])	△ 5.6‐10 mmol/L	△ % time < 3.5 mmolL	△ % time > 10 mmol/L		90% happy to have automated control 95% would recommend to a friend or family member if admitted to hospital	None (one adverse event not related to study)	Single‐arm study Sample size Study duration Intention to treat analysis
					+ 21.8% (95% CI 10.4‐33.1) *P* = ·0004	No significant change Control 0 (IQR 0‐2.7), HCL 0 (IQR 0‐0.4) *P* = .35	−19.0% (95% CI −4.7 to −33.3) *P* = .011				
Bally et al. (2018)^124^	3 mo 136 adults with T2D on insulin UK and Switzerland Randomized trial HCL (*n* = 70) versus SC insulin Inpatient hyperglycaemia requiring SC insulin	FCL Dana‐R with MPC algorithm	Two‐centre multi‐national inpatient, open‐label study comparing HCL with standard insulin therapy	△ % Time in target range (5.6‐10 mmol/L [100‐180 mg/dL])	△ 100–180 mg/dL	△ % time < 54 mg/dL	△ % time > 180 mg/dL	No change TDD insulin	87% happy to have automated control 98% would recommend the system to a friend or family member if admitted to hospital	Nil serious 3 FCL group; 2 sensor failure and 1 pump check error 3 control; bruising at cannula site and skin irritation	Study duration
					+24.3 ± 2.9 (95% CI 18.6‐30.0) *P* < .001	No significant change *P* = .80	−25.9 ± 3.4 (95% CI −19.2 to −32.7) *P* < .001				
Boughton et al. (2021)^84^	20 d × 2 26 adults with T2D requiring dialysis for 6 y) United States Randomized crossover trial ESRF on peritoneal‐ or haemo‐dialysis	FCL CamAPS HX	Open‐label, multinational, two‐centre study to evaluate safety and efficacy of FCL versus standard therapy	△ % Time in target range (5.6‐10 mmol/L [100‐180 mg/dL])	△ 5.6‐10 mmol/L	△ % time <3.9 mmol/L	△ % time > 10 mmol/L		More personal time and freedom improved peace of mind and reassurance Device burden (discomfort, wearing pump, carrying phone) were the most common limitations	One severe hypo‐glycaemia during closed loop	Single‐arm study Sample size Study duration
					15.1% (95% CI 8.0‐22.2) *P* < .001	Reduced to 0.12 (IQR 0.02‐0.44) from 0.17 (IQR 0.00‐1.11) *P* = .04	Reduced from 56.6 ± 25.1 to 42.6 ± 14.3 *P* = .003				
Daly et al. (2023)^85^	8 wk × 2 26 adults with T2D United States Randomized crossover trial HbA1c < 12% (< 108 mmol/L)	FCL CamAPS HX	Open‐label, single‐centre, randomized crossover study to determine the benefits of FCL systems in adults with T2D	△ % Time in target glucose range (3.9‐10.0 mmol/L [70‐180 mg/dL])	△ 5.6‐10 mmol/L	△ % time <3.9 mmol/L	△ % time > 180 mg/dL		Hypoglycaemia confidence scores and PAID scores similar between interventions Hypoglycaemia worry score higher following closed‐loop (15.0 [IQR, 6.5‐20.0]) versus a median of 9.5 (IQR, 6.0‐21.0)	One serious adverse event related to study procedure; abscess at pump site needing incision and drainage	Single‐arm study Sample size Study duration Intention to treat analysis
					+35.3 (95% CI 28.0‐42.6) *P* < .001	No significant change Closed loop 0.44 (IQR 0.19‐0.81) Control 0.08 (IQR, 0.00%‐1.05%) *P* = .43	−35.2 (95% CI, −42.8 to −27.5) *P* < .001				
CFRD											
Scully et al. (2022)^120^	3 mo 13 adults and adolescents with CFRD (age 15‐64.6 y) on MDI or insulin pump United States Retrospective analysis Using Dexcom CGM	HCL Tandem T:Slim × 2 with Control IQ	Multicentre retrospective observational study assessing outcomes post‐HCL initiation at 1 and 3 mo	△ % time in target range 70‐180 mg/dL	△ 70‐180 mg/dL	△ % time < 54 mg/dL	△ % time > 250 mg/dL		Not assessed	Not reported	Sample size
					69.5 ± 5.1 at 3 mo from 54.3 ± 5.1 *P* = .001	0.3 ± 0.1 at 3 mo from 0.2 ± 0.1 at baseline *P* = .349	8.6% ± 4.6% from 21.1% ± 4.5% at baseline *P* = .008				
Sherwood et al. (2020)^121^	1 wk 3 aged ≥ 18 y with CFRD and United States Pilot TDD insulin ≥ 0.1 u/kg/d	HCL or bi‐hormonal closed loop	Three‐arm, random‐order, crossover pilot study comparing HCL, BH closed loop and SC	△ mean CGM △ % time below 3 mmol/L	△ 70‐180 mg/dL	△ % time < 54 mg/dL	△ % time > 250 mg/dL	Mean CGM glucose mg/dL SC 159 ± 35 HCL 149 ± 10 BH 139 ± 15	Management burden and time spent thinking about diabetes reduced with closed loop. Freer with food choices and overall greater peace of mind		Small sample size
					SC 62 ± 23 HCL 76 ± 9 BH 80 ± 10	SC 0.3 ± 0.3 HCL 0.5 ± 0.1 BH 0.2 ± 0.2	SC 9 ± 7 HCL 5 ± 2 BH 4 ± 4				

*Note*: Changes to % times in range are represented as either mean ± SD, mean (95% CI) or median (IQR).

Abbreviations: AID, automated insulin delivery; BH, bi‐hormonal; CFRD, cystic fibrosis‐related diabetes; CGM, continuous glucose monitor; CI, confidence interval; DIDS, diabetes impact and device satisfaction scale; ESRF, end‐stage renal failure; FCL, fully closed‐loop; HCL, hybrid closed‐loop; IQR, interquartile range; MDI, multiple daily injections; MPC, model predictive control; PAID, problem areas in diabetes scores; PROMIS, patient‐reported outcomes measurement information system; SC, standard care; SD, standard deviation; TDD, total daily dose; TIR, time in range; T2D, type 2 diabetes.

## 6 MAXIMIZING OPPORTUNITIES

### 6.1 Form factor

HCL systems with a secondary handset (smartphone or controller) interface may be more easily operated by those with limited dexterity or vision. However, these devices might lack the specific algorithmic features the user desires.

Similarly, the physical attributes of certain HCL systems may garner increased interest and demand from people with diabetes, irrespective of algorithmic performance; and may explain the rising popularity of the first tubeless HCL system, the OP5. The limited reservoir size of the OP5 may limit its practical use to those with lower daily insulin demands, because individuals with very high daily insulin requirements need to change their pods more frequently, or use more concentrated (U200) insulins (with attendant potential risks).

### 6.2 Financial

Clinical trial and real‐world data led to a technology appraisal of HCL systems for pwT1D in England and Wales at the end of 2023 where a phased implementation of HCL systems to assist in the management of T1D for adults with disabling hypoglycaemia or suboptimal control despite best current management and all children and young people has been mandated within a publicly funded healthcare system.^125^ Out‐of‐pocket costs in European countries vary, and in the United States, where healthcare is insurance based, consensus guidelines recommend that the use of technology should be ‘individualized based on a patient's needs, desires, skill level, and availability of devices’^126^; individual out‐of‐pocket costs are determined by the presence and extent of insurance coverage.

### 6.3 Deprivation

National pump audit data in the UK from 2017 to 2018^127^ and from 2019 to 2020^128^ reports an inverse relationship between the use and access to insulin pump therapy with socioeconomic quintile, and in T1D care, lower socioeconomic status is associated with poorer diabetes‐related outcomes.^129^, ^130^, ^131^ With RWE demonstrating that in adults with T1D, access to technology attains equal improvements in glycaemia, irrespective of socioeconomic or ethnic background,^132^, ^133^ it is hard to refute a role for inequities in access to technologies as a contributor to the poorer diabetes outcomes observed in those with T1D with lower socio‐economic status.

### 6.4 Education

For patients starting a system, informative discussions with HCPs regarding system suitability and effective troubleshooting are only possible if those HCPs are familiar with available commercial systems; adequate training for HCPs to achieve and maintain this level of knowledge is crucial. Compared with their more affluent counterparts, patients from the most socioeconomically deprived backgrounds report that HCPs display a reduced awareness of available technologies during their discussions with them.^134^ Confronting the issue of HCP bias to challenge inequities is an area that HCP training can also address. For those living with diabetes, multilingual educational documents (and resources for those with additional hearing, visual, literacy or numerical processing needs) can help to broaden access, and remote training platforms may assist with adapting to and making the best use of newer devices.^135^

## 7 THE FUTURE

### 7.1 Single hormone fully closed‐loop systems

Single hormone fully closed‐loop (SHFCL) systems utilize adaptive algorithms that do not require mealtime announcements. A number of small, controlled inpatient^123^, ^124^ and outpatient^84^, ^85^ crossover trials have shown the ability of such systems to enhance glycaemic control without increasing the risk of hypoglycaemia in individuals with T2D (Table 2). The safety and efficacy of a SHFCL system in the outpatient setting during 8 weeks of unrestricted living in 26 adults with T1D has also recently been shown, with improved glycaemia and no severe hypoglycaemia or ketoacidosis.^136^

### 7.2 Dual hormonal systems

The outcomes of RCTs assessing the efficacy and safety of dual hormone (DH) systems, largely based on insulin and glucagon, in pwT1D, were recently evaluated in a meta‐analysis by Zeng et al.^102^ The short studies suggest non‐inferiority compared with HCL, with practical considerations that also need to be considered. The DARE study, a 12‐month multicentre RCT in the Netherlands including 240 adults with T1D that aims to determine the glycaemic and QoL impacts and cost efficacy of DH fully closed‐loop (DHFCL) systems compared with usual care (HCL or SAP), is currently underway.^137^ Outcomes from this longer duration study may shed light on the comparative clinical efficacy and practicalities of DHFCL systems versus standard care undetected by shorter, smaller studies.

### 7.3 Sensing different metabolites

Wolkowicz et al.^138^ reviewed important analytes that could serve as added inputs to enhance closed‐loop control in T1D. Incorporation of insulin sensing to provide a true measure of insulin‐on‐board, and the integration of a combined continuous glucose–ketone monitor, were among those suggested. Further research to assess the accuracy of analyte detection, reliability of communications to control algorithms and the cloud, and the potential for multianalyte‐detecting sensors to enhance wearability are needed before broader adoption can be considered.

### 7.4 Additional devices

DeBoer and colleagues showed that use of the heart rate as an additive input to AID systems via a heart rate monitor triggering an exercise algorithm for glycaemic control reduced the time spent below range during exercise in 18 adolescents with T1D compared with standard HCL systems.^139^

Alerts from an AI‐controlled smartwatch app that detects eating behaviour has been shown to improve glycaemic control in pwT1D not using AID systems with a history of missed or late boluses.^140^ Communication between such applications and a HCL system could improve the accuracy of automated boluses, while communication from the HCL algorithm to smartwatch interface could provide user alerts of automated mealtime boluses; this has the potential to reduce incidences of manual postprandial boluses and subsequent hypoglycaemia risk.

### 7.5 Artificial intelligence

AI is increasingly integrated into healthcare data applications. ML is a subset of AI that allows a computer system to learn from experience rather than explicit programming. ML uses an algorithm to analyse and learn from large amounts of data. ML then makes decisions based on its learning to process unseen datasets. ML algorithm performance increases with the amount and diversity of data to which it is exposed.^141^ A recent pilot feasibility RCT tested the concept of ML to replicate the MPC algorithm in 15 pwT1D.^86^ Outcomes showed that ML was able to produce equivalent outcomes with a 6‐fold lower computational demand. Results indicate that the use of AI in algorithmic insulin delivery can enhance the battery life of the typically limited‐power devices in which the control algorithms are commonly situated. Simultaneously, reduced processing power may increase the number of devices that can function as HCL system interfaces.

### 7.6 Bluetooth

Bluetooth technology is continually advancing. The earliest form of BLE was Bluetooth 4.0, which was launched in 2010. The faster and more powerful Bluetooth 5.0 was released in 2016, with the most recent Bluetooth 5.4 introducing improvements that include periodic advertising and enhanced encryption, offering security and energy‐efficiency advantages.

In 2022, Bluetooth announced a new Bluetooth audio broadcast function called Auracast.^142^ If integrated to HCL systems, audio‐controlled insulin delivery could increase the accessibility of systems to those with challenges to literacy or vision. The next major iteration of Bluetooth is Bluetooth 6.0. Details of this system have yet to be released, but it will probably continue improving energy efficiency, security, communication range and speed. Key in upgrades to existing HCL systems is the integration with advancing forms of wireless connectivity to optimize performance.

## 8 WHAT NEEDS TO BE IMPROVED?

### 8.1 Interoperability

**FIGURE 4:**
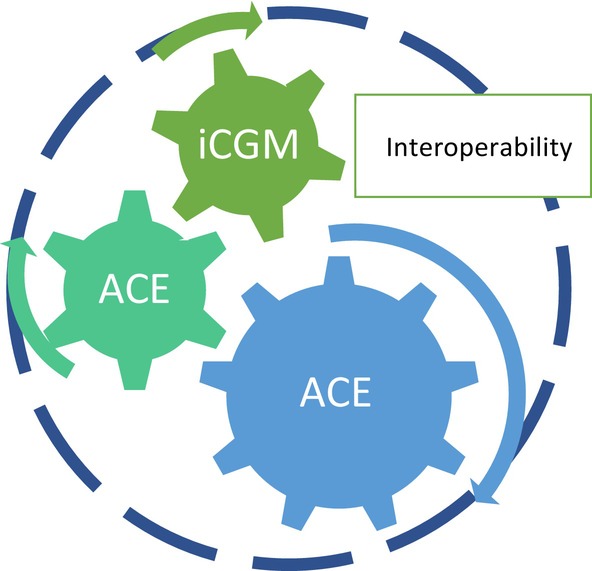
Cogs in the wheel of interoperability; FDA designations of iCGM, ACE and iAGC statuses have defined regulatory milestones that need to be met before true interoperability between commercial HCL systems can be achieved. ACE, alternate controller‐enabled; FDA, US Food and Drug Administration; HCL, hybrid closed‐loop; iAGC, interoperable automated glycaemic controller; iCGM, integrated continuous glucose monitoring.

With respect to commercial HCL systems, interoperability refers to approved connection between various commercial insulin pumps with CGM devices. It does not refer to adversarial interoperability, otherwise known as competitive compatibility, which is the communication between systems despite the resistance of one or more of the connecting systems.^143^ Adversarial interoperability allows OS algorithms to connect to a number of commercial insulin pumps and CGM devices, facilitating user choice and personalization (Table 1).^40^ In the commercial market, interoperability could offer the same benefits but necessitates commercial agreements between device manufacturers and overcoming regulatory barriers. In August 2017, the JDRF launched the Open Protocol Initiative, aiming to assist in the delivery of interoperable diabetes technologies.^144^ This was followed by a series of US Food and Drug Administration (FDA) regulations to ensure the safety of such systems^145^ (Figure 4).

In 2018, the FDA defined the ‘integrated continuous glucose monitor’ (iCGM) that became the first cog in the wheel towards interoperability^146^ iCGM status is attained when a CGM device meets the FDA's criteria for accuracy and safety. Currently, the Dexcom G6,^147^ Libre 2, Libre 3 and Eversense^148^ devices have iCGM status. In 2019, the FDA defined the ‘alternate controller‐enabled’ (ACE) pathway^145^, ^149^ that became the second cog in the wheel towards interoperability; ACE status is attained when an insulin pump can work safely with multiple algorithms. Tandem's t:slim X2 was the first insulin pump to gain ACE status. In 2020, the FDA defined the ‘interoperable automated glycaemic controller pathway’,^145^, ^150^ the third and final cog in the wheel towards interoperability; interoperable automated glycaemic controller (iAGC) status is attained when an algorithm can communicate with a number of different insulin pumps and CGM devices.

One of the most commonly used versions of OS AID is ‘Loop’ (Table 1). In 2018, the not‐for‐profit organization, Tidepool, which aims to deliver OS AID technologies, officially launched ‘Tidepool Loop’, a project dedicated to ‘delivering an officially supported, FDA regulated version of the Loop app’.^151^ Designation as an iAGC system was a clear aim for Tidepool Loop.^145^ In January 2023, Tidepool Loop was granted FDA approval^152^ and became the first OS‐derived algorithm to achieve this status.^153^ The commercial launch of the FDA‐approved HCL system incorporating Tidepool Loop as the twiist AID system is awaited.

### 8.2 Open standards for AI

Open standards are rules allowing any user to create compatible and consistent products^154^ and can assist with AI‐assisted algorithmic insulin delivery by establishing standards that developers must adhere to. Such standards address issues such as data security, privacy and ownership. The presence of open standards facilitates increased transparency in the processes of algorithm creation. This was probably to improve trust in, and thus enhance the utilization of such systems. Increased use will allow increased data exposure that could facilitate the development of more advanced algorithms. Open standards may also facilitate interoperability.^155^

An initiative called STANDING Together has released new international consensus standards that aim to balance the benefits of AI in healthcare against the risks of algorithmic bias and harm to those under‐represented in healthcare datasets.^156^, ^157^ These are especially relevant to diabetes technologies^158^, ^159^, ^160^ and diabetes research that^161^ are less accessible to and less representative of those from minority backgrounds.

## 9 CONCLUSIONS

Establishing connectivity between insulin pumps and CGM devices has enabled significant improvements in diabetes care. The emergence of AID systems, particularly HCLs, has had a significant and positive impact on the lives of people living with T1D. The evidence presented here highlights the potential benefits that these devices can deliver at a population level. Underpinning the success of current AID systems has been Bluetooth connectivity. Advances in engineering will enable better connectivity, which may lead to improved diabetes devices and user experience. It may also enable opportunities to consider connectivity between different combinations of devices and integrate data from different sources for improved outcomes. Future work may overcome the challenges in connectivity and interoperability and define standards for AI and information exchange between diabetes devices.

## AUTHOR CONTRIBUTIONS

AA and SH have equally contributed to the design, draft and review of the article. Both AA and SH have approved the version of the article to be published.

## FUNDING INFORMATION

SH is a recipient of the Medical Research Council Clinical Academic Partnership award (MR/W030004/1). AA is supported by an unrestricted educational grant from Abbott UK, awarded to SH.

## CONFLICT OF INTEREST STATEMENT

AA has no conflicts of interest to declare. SH has served on advisory boards for Tandem, Dexcom and Medtronic; has undertaken non‐promotional educational and/or consultancy work for Abbott UK, Insulet, Dexcom and Roche; and has received an unrestricted educational research grant from Abbott UK and an investigator‐initiated research grant from Insulet.

### PEER REVIEW

The peer review history for this article is available at https://www.webofscience.com/api/gateway/wos/peer-review/10.1111/dom.15920.

## Data Availability

Data sharing not applicable to this article as no datasets were generated or analysed during the current study.
